# A case series of intraosseous hemangioma of the jaws: Various presentations of a rare entity

**DOI:** 10.4317/jced.54285

**Published:** 2017-11-01

**Authors:** Srinivasa R. Chandra, Eleanor Chen, Timothee Cousin, Dolphine Oda

**Affiliations:** 1 MD, DDS, Clinical Assistant Professor, 1959 NE Pacific St, Department of Oral & Maxillofacial Surgery, University of Washington School of Dentistry, Seattle, Washington 98195-7133, USA; 2 MD, PhD, Assistant Professor, Department of Pathology, University of Washington Medical Center , 1959 NE Pacific St Box 357705; 3 DDS, Candidate, Class of 2017, 1959 NE Pacific St, Room D322, University of Washington School of Dentistry, Seattle, Washington 98195, USA; 4 BDS MSc, Professor, 1959 NE Pacific St, Department of Oral & Maxillofacial Surgery, University of Washington School of Dentistry, Seattle, Washington 98195-7133, USA

## Abstract

**Background:**

Hemangiomas of the soft tissue are common in the head and neck area, especially in the tongue and in children under ten years of age. Intraosseous hemangiomas of the mandible and maxilla (IHM), on the other hand, are exceedingly rare and are not well characterized. This study presents six IHM cases focusing on the clinical, radiographic, and histologic characteristics.

**Material and Methods:**

Six cases of IHM were retrieved from the archives of the Biopsy Services at the University of Washington. Clinical, radiologic, and histologic findings are described.

**Results:**

A total of six cases of IHM were reviewed. The patient age range was 16 to 65; the group comprised three females and three males. All six cases presented as swellings, two caused tooth resorption, and one was associated with pain and numbness. Three of the six IHM were present in the body of the mandible, two in the area of the extracted right mandibular third molar, and one in the anterior maxilla between the right canine and lateral incisor. Radiographically, five were radiolucent and one was radiopaque. Of the five radiolucent, two were unilocular and three multilocular. The one radiopaque case was exophytic, simulating a large osteoma. Histologic features ranged from cavernous to a mix of venous and arterial types. Follow-up is available for all six cases ranging between one to seven years; only one case recurred within the first year post-surgery.

**Conclusions:**

IHM are exceedingly rare; IHM do not present in a consistent manner both clinically and radiographically. It is therefore important to recognize the wide spectrum of IHM’s clinical, radiographic, and histological presentations.

** Key words:**Hemangioma, Vascular Malformation, mandible, maxilla.

## Introduction

Hemangiomas are benign vascular tumors composed of endothelial cells and supporting cells that line blood vessels. Subsequent to the 1996 ISSVA classification of vascular lesions, it has been suggested that the term “hemangioma” was used erroneously in describing soft tissue vascular lesions and should be discarded in favour of two main types of vascular anomalies: Vascular tumors and vascular malformations (1). However, ISSVA classification has not been extended to osseous vascular lesions (2). Since the biological nature of these lesions is still controversial, we frequently refrain from defining them by any one category and refer to them as “benign vascular lesions” (3).

Soft tissue hemangiomas are common, especially in children under the age of ten (4-6). Intraosseous hemangiomas however, especially those of the mandible and maxilla (jaws) (IHM), are exceedingly rare (7). In other parts of the body, intraosseous hemangiomas comprise less than 1% of all hemangiomas (3,8) and occur more commonly in the vertebrae and craniofacial bone, including the skull and the jaws, followed by long bones (9-12). Women are slightly more susceptible to developing intraosseous hemangiomas (5,11,13,14). Regarding the jawbone, the mandible is affected three times as commonly as the maxilla (13). In the mandible, the body is the more common location, while the posterior mandible, the ramus, and the condyle are also sites affected (14). IHMs tend to occur in the first three decades of life,13 while that of other bones tends to occur in older patients with peak incidence in the fifth decade and an age range of two to 85 years (9,11,12). Clinically and radiographically, IHM can be a diagnostic challenge since a wide range of presentations has been reported. In this study, we present six cases of IHM, diagnosed between 2008 and 2015, with the objective of highlighting the variability in IHM’s clinical, radiographic, and histologic features.

## Case Reports

The radiographic and clinical findings of six cases of intraosseous hemangioma of the maxilla and mandible were reviewed by an oral surgeon (SC), and the hematoxylin and eosin (H&E) stained glass slides were independently reviewed by two pathologists (DO&EC). Statistical testing to determine any significant difference in our comparisons was not possible due to the small size of our series. IRB approval was obtained to perform this study.

-Clinical Findings

**Figure 1:**
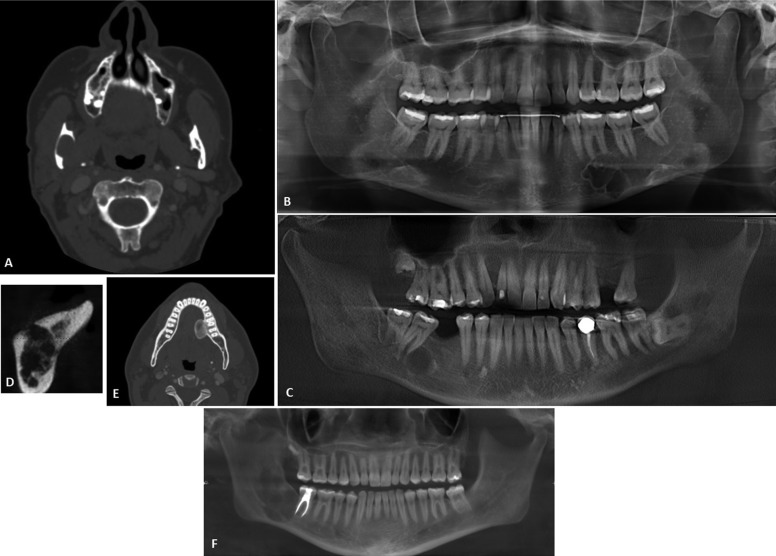
Radiological features of Intraosseous Venous Malformation of the Mandible and Maxilla. A: This CT scan image demonstrates a unilocular expansile radiolucency at the right posterior mandible in area of extracted tooth #32. B: This panoramic image demonstrates a multilocular expansile radiolucency in the left body of mandible between teeth #18 and #20. Note apical resorption of tooth #19. C: This is a portion of a panoramic radiograph demonstrating a unilocular expansile radiolucency in the right maxilla between teeth #6 and #7, which is pushing them apart. D: This CT scan image demonstrates a multilocular and mildly expansile radiolucency in the right body of the mandible in an edentulous area. E: This panoramic image demonstrates a large radiopaque expansile nodule in the lingual left body of the mandible protruding into the floor of mouth simulating a large osteoma. F: This panoramic image demonstrates a large and multilocular expansile radiolucency in the right posterior mandible in area of extracted tooth #32 extending posteriorly into the ramus. Note distal root resorption of tooth #31.

Of the 35,320 biopsies reviewed at the University of Washington Biopsy Services between the years 2008 and 2015, six cases were diagnosed with intraosseous hemangioma (an incidence of 0.017%).  Table 1 summarizes the main clinical, radiographic, and histologic features of the six cases. There were three males and three females with an age range of 16 to 65 years (mean = 41). The age range of the three males was 28 to 46 years and of the three females it was 16 to 65 years. Five of the cases were in the mandible and one in the maxilla (case 3; Fig. 1C). In the mandible, three cases (cases 2, 4, and 5; Fig. 1B,D, E) were in the body of the mandible, and two were in the posterior mandible in area of the right mandibular third molar extending posteriorly into the ramus (cases 1 and 6; Fig. 1A,F). Five of the six cases were mildly expansile, while case five was significantly expansile with lingual extension into the floor of mouth (Fig. 1E). Five cases reported no clinical symptoms other than the expansion/swelling. Case four (Fig. 1D) reported pain followed by numbness and a mild expansion in an edentulous area. Cases one and six (Fig. 1A,F) were in the area of the right mandibular third molar extending to the ramus. However, neither of these cases reported symptoms of pain or numbness.

**Table 1:**
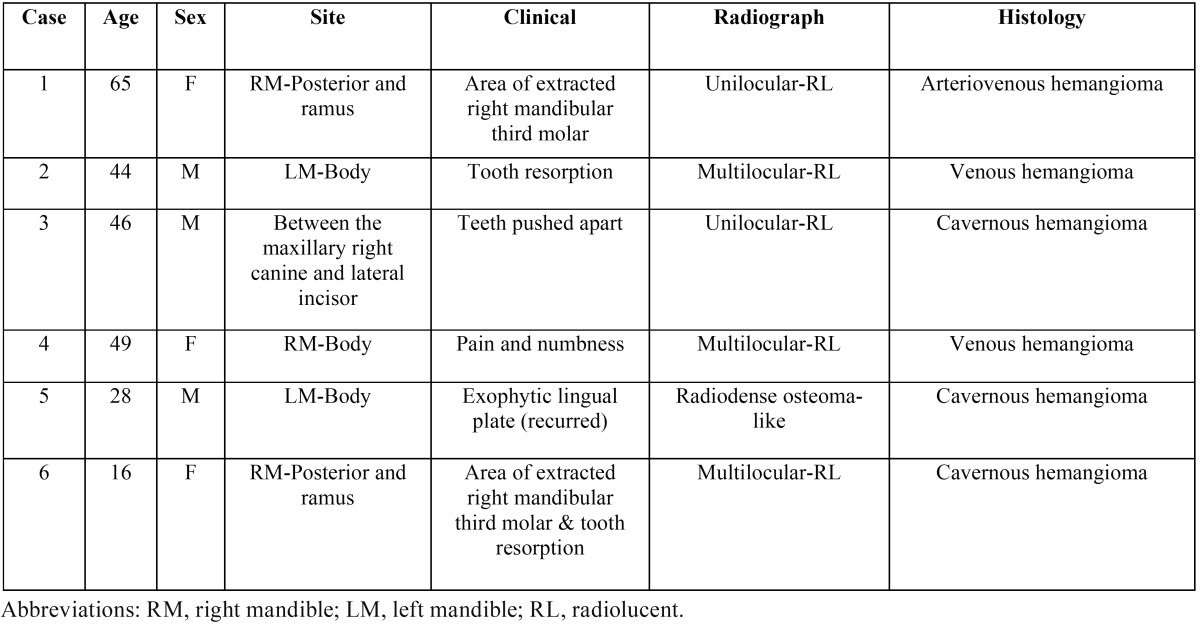
Summary of Clinical, Radiographic, and Histologic Features of Six Cases of Intraosseous Hemangioma of the Mandible and Maxilla.

-Radiographic Findings

There is a wide range of radiographic presentations; five of the six cases were radiolucent (Fig. 1A-D,F), while case 5 was radio-paque (Fig. 1E). Three of the radiolucent cases were multilocular (cases 2, 4, and 6; Fig. 1B,D, F), and two were unilocular (cases 1 and 3; Fig. 1A,C). Cases 2 and 6 were associated with tooth resorption: the left mandibular first molar in case 2 and the right mandibular second molar in case 6 (Fig. 1B,E). The tumor in case 3 expanded and pushed the right maxillary lateral incisor and canine apart. Case 5 was completely radiopaque (Fig. 1E) and presented as an exophytic lesion protruding lingually into the floor of mouth. All six cases were isolated lesions.

-Pathologic Findings

**Figure 2:**
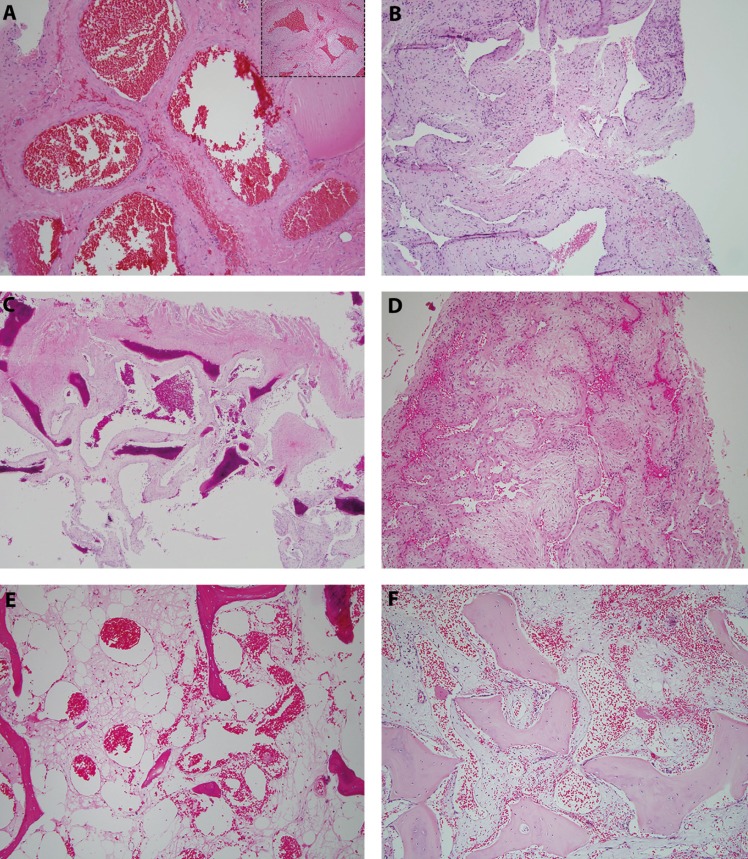
Histological features of Intraosseous Venous Malformation of the Mandible and Maxilla. A: Vascular spaces filled with erythrocytes and lined by one layer of flat endothelial cell. The vascular spaces represent veins and arteries (inset: mainly arteries) (H&E stain, x100). B: Vascular spaces intertwining and lacking erythrocytes. They are lined by one layer of flat endothelial cells. The vascular spaces represent veins (H&E stain, x100). C: Large vascular spaces filled with erythrocytes and lined by one layer of flat endothelial cells. They are separated by strands of fibrous connective tissue and bony trabeculae (H&E stain, x100). D: Venous-type vessels coalescing with each other. They are lined by one layer of flat endothelial cells and have sparse amount of erythrocytes. The vascular spaces represent veins (H&E stain, x100). E: Large vascular spaces of variable sizes filled with erythrocytes and lined by one layer of flat endothelial cells. The spaces are separated by fibrous connective tissue and bony trabeculae (H&E stain, x100). F: Large vascular spaces of variable shapes partially filled with erythrocytes and lined by one layer of flat endothelial cells. They are separated by bony trabeculae (H&E stain, x100).

The H&E-stained sections were reviewed by two independent pathologists (DO and EC) for a consensus of histologic features. Three were cavernous hemangiomas, two were venous hemangiomas, and one was an arteriovenous hemangioma. All six cases manifested large vascular spaces, four of the cases (cases 1, 3, 5, and 6; Fig. 2A,C,E,F) showed abundant erythrocytes within the vascular spaces. Case four (Fig. 2D) showed spaces with sparse erythrocytes, and case two (Fig. 2B) showed spaces with little to no erythrocytes, likely reflecting varying degrees of circulatory stasis. Three of the cases were cavernous hemangiomas (cases 3, 5, and 6). All cavernous hemangiomas (cases 3,5, and 6, Fig. 2C,E, F) were made up of cystically dilated spaces, filled with erythrocytes and separated by dense connective tissue in some areas and by strands of bony trabeculae in other areas (Fig. 2C,E,F). Cases two and four (Fig. 2B,D) were made up of very large and tortuous vascular spaces lined by one layer of flat endothelial cells with sparse erythrocytes. Case one (Fig. 2A) had a mixture of arterial and venous channels lined by one layer of flat endothelial cells and a muscular wall of varying thickness, separated by thick strands of connective tissue. The venous hemangiomas (cases 2 and 4; Fig. 2B,D) demonstrate large-caliber vessels with thin-walled and collapsible lumina. In contrast, the arterial vessels in case one (Fig. 2A) of arteriovenous hemangioma showed thick muscular walls with smaller lumina.

## Discussion

In general, intraosseous hemangiomas are rare, constituting less than 1% of all hemangiomas (3,8) and most commonly occur in the vertebrae followed by the skull bones (9-12). IHM, on the other hand, are exceedingly rare (7). They are also clinically and radiographically challenging to diagnose (5,7,8,14,15). This study presents six cases of IHM exhibiting variability in clinical and radiographic findings and several distinct histologic patterns, providing more insight into the behaviour of this rare condition.

Clinically, intraosseous hemangiomas of the bones in general and that of the jaws tend to occur slightly more commonly in females with a ratio of 3:2 female to male (12). Our series of six cases consists of an equal distribution of males and females ( Table 1). The peak age for intraosseous hemangiomas in general is in the fifth decade (10-12). The age range for IHM is reported in the literature to be in the first three decades of life (13). In contrast, our case series demonstrates a wide age range of 16 to 65 with a mean age of 41. The mandible has been reported as a more common site for IHM with a mandible to maxilla ratio of 3:1 (13). Our series shows a predominant occurrence in the mandible (five out of six cases with a ratio of 5:1). All five cases of the mandible affected the body and the posterior mandible to ramus, consistent with the distribution as previously reported in the literature (10,14,16). Regarding clinical symptoms, five of the six cases presented with mild expansion consistent with what is reported (7-14). Only case five (Fig. 1E) showed significant expansion. Five of the six cases had no symptoms including pain and anesthesia, and only one presented with pain followed by numbness. The lack of clinical symptoms of pain and anesthesia is consistent with what is reported of all bone hemangiomas including IHM (7-14). Five of the six cases were associated with teeth, and only one occurred in an edentulous area.

Radiographically, five of the six cases presented as radiolucent lesions, which is consistent with that reported in the literature (3,7,8,9,16,17). Most of intraosseous hemangiomas are radiolucent with a wide range of radiographic presentations, including the more common multilocular/honeycomb appearance to unilocular, ‘spider-web’ or sunray appearance (7,8,9,17). Of the five radiolucent cases in the current series, three were multilocular and two were unilocular fitting the radiolucent spectrum as reported. However, the radiopaque presentation of the intraosseous hemangioma is rare; case five (Fig. 1E) had a unique presentation of a radiopaque nodule simulating an osteoma. This osteoma-like presentation is rare and unique to this report. Radiologically opaque hemangiomas of the jaw are reported but mainly to have features of ground-glass appearance simulating fibrous dysplasia (17).

Histologically, all six cases were diagnosed based on morphologic features present on the H&E–stained slides, three of the six cases were interpreted as cavernous hemangioma (Fig. 2C,E,F). Both cavernous and mixed arteriovenous patterns of hemangiomas are common (8). In our study, four of six cases demonstrated either cavernous or mixed arteriovenous patterns. Two cases in our series (cases 2 and 4) consisted of predominantly venous-type vessels, which is an unusual histologic pattern for hemangiomas.

All six cases were conservatively surgically excised, and only one recurred with a follow-up period of one to seven years. Case 5 recurred within one year of excision but has not recurred since the last excision.

In conclusion, we present six cases of IHM with an overall benign clinical outcome, except for one case which recurred within one year of treatment. This recurrence suggests that although hemangiomas of the jaw are benign, they have a small potential for recurrence. Importantly, IHM do not present in a consistent manner both clinically and radiographically. It is therefore important to recognize the wide spectrum of IHM’s clinical, radiographic, and histological presentations.
